# Time from dementia diagnosis to nursing-home admission and death among persons with dementia: A multistate survival analysis

**DOI:** 10.1371/journal.pone.0243513

**Published:** 2020-12-04

**Authors:** Marit Mjørud, Geir Selbæk, Espen Bjertness, Trine Holt Edwin, Knut Engedal, Anne-Brita Knapskog, Bjørn Heine Strand

**Affiliations:** 1 Norwegian National Advisory Unit on Ageing and Health, Vestfold Hospital Trust, Tønsberg, Norway; 2 Department of Geriatric Medicine, Oslo University Hospital, Oslo, Norway; 3 Faculty of Medicine, Institute of Clinical Medicine, University of Oslo, Oslo, Norway; 4 Faculty of Medicine, Department of Community Medicine and Global Health, Institute of Health and Society, University of Oslo, Oslo, Norway; 5 Faculty of Medicine, Institute of Health and Society, University of Oslo, Oslo, Norway; 6 Department of Chronic Diseases and Ageing, Norwegian Institute of Public Health, Oslo, Norway; Ehime University Graduate School of Medicine, JAPAN

## Abstract

**Objectives:**

To estimate transition times from dementia diagnosis to nursing-home (NH) admission or death and to examine whether sex, education, marital status, level of cognitive impairment and dementia aetiology are associated with transition times.

**Design:**

Markov multistate survival analysis and flexible parametric models.

**Setting:**

Participants were recruited from the Norwegian Registry of Persons Assessed for Cognitive Symptoms (NorCog) in specialist healthcare between 2008 and 2017 and followed until August 2019, a maximum of 10.6 years follow-up time (mean 4.4 years, SD 2.2). Participants’ address histories, emigration and vital status were retrieved from the National Population Registry from time of diagnosis and linked to NorCog clinical data.

**Participants:**

2,938 home-dwelling persons with dementia, ages 40–97 years at time of diagnosis (mean 76.1, SD 8.5).

**Results:**

During follow-up, 992 persons (34%) were admitted to nursing-homes (NHs) and 1,556 (53%) died. Approximately four years after diagnosis, the probability of living in a NH peaked at 19%; thereafter, the probability decreased due to mortality. Median elapsed time from dementia diagnosis to NH admission among those admitted to NHs was 2.28 years (IQR 2.32). The probability of NH admission was greater for women than men due to women´s lower mortality rate. Persons living alone, particularly men, had a higher probability of NH admission than cohabitants. Age, dementia aetiology and severity of cognitive impairment at time of diagnosis did not influence the probability of NH admission. Those with fewer than 10 years of education had a lower probability of NH admission than those with 10 years or more, and this was independent of the excess mortality in the less-educated group.

**Conclusion:**

Four years after diagnosis, half of the participants still lived at home, while NH residency peaked at 19%. Those with fewer than 10 years of education were less often admitted to NH.

## Introduction

Dementia disorders are typically progressive, leading to dependency and death [1–3], and progression of dementia is associated with numerous factors [4–6]. Prognosis is of key importance for newly diagnosed persons with dementia and their families, especially in regard to the amount of time to dependency and the need for around-the-clock assistance. Factors predicting the amount of time to nursing home (NH) admission may differ between persons assessed for cognitive decline in primary healthcare compared to those assessed in specialist healthcare [7–9]. Furthermore, NH admission may depend on the quality and quantity of health and social services provided to persons in their homes [10,11], which may differ among countries.

Healthcare in Norway, including both domestic nursing and nursing homes (NHs), is a public service financed through the tax system. Approximately 9% of nursing homes are run by for-profit or non-profit private organizations, but there are no differences between admission to public vs private institutions, as the municipalities pay for the stay in the private institutions. Everyone in need of long-term care will be offered such assistance either at home or in a NH, depending on the individual’s degree of dependency and family situation. Even with frequent domestic nursing care, perhaps as much as 6 visits a day/night, persons with moderate or severe dementia might need to move to a nursing home, if or when the symptoms become too severe, or if the informal caregiver is exhausted. More than 80% of persons living in Norwegian NHs today have dementia [12], and the average length of stay for this group is reported to be 2.4 years [13], similar to figures from the Netherlands [14], where the median length of NH stay for persons with dementia was found to be 2.5 years. Figures on the general NH population in European countries show a mean length of stay of approximately 2 years [14–16].

Several studies on time to NH admission have been conducted, examining nursing-home placement at first follow-up [17], or admission during a 6-month period [10], including persons with Alzheimer’s disease [18–20] or comparing different diagnostic groups, like Lewy body dementia and Alzheimer’s disease [21]. A Dutch study found the median elapsed time from diagnosis to NH admission to be 3.9 years [14], and another European study found that ~20% of home dwelling persons with Alzheimer’s disease were institutionalized within 36 months from baseline assessment [18]. Other studies examines how various interventions effect time to admission, like use of dementia drugs [20], or informal carer-training [22]. Few however, examine time from diagnosis to NH or death, and information on transition times is scarce.

Both time to dependency and time to mortality in persons with dementia are associated with older age, poorer cognition and reduced functioning in activities of daily living (ADL) at the time of diagnosis [1,4–6,23–25]. As functional and cognitive impairment progress during the course of dementia, support at home provided by formal carers or family members may delay NH admission [5,10,26]. Studies have indicated that married persons or persons with adult children are less likely to be admitted to NHs, probably because of the support they receive at home [10,27–31]. In addition, cohabitation may extend life expectancy after a dementia diagnosis [4].

Increased impairment in both cognition and personal activities of daily living are associated with a greater risk of NH admission [5,6,23,25,27,32]. Compared to persons with dementia due to Alzheimer’s disease (AD), persons with non-AD dementia, particularly Lewy body dementia, have been found to experience earlier admission to NH [21,22,33] as well as a shorter life expectancy [1,24,33].

In persons with dementia, the effect of educational level on time to NH admission is unknown [34], and its effect on mortality is equivocal [23,35]. In general, persons with more education experience better health and functioning [36], with a trend towards greater access to more-specialized treatments, at least in Norway [37,38].

In order to plan and improve future healthcare services for people with dementia, information on time to NH admission and associated risk factors is important for policy makers but just as critical for persons with dementia and their families. Hence, the twofold aim of this study was to estimate transition times from dementia diagnosis to nursing-home admission or death and to examine whether background factors such as sex, education, marital status, level of cognitive impairment and dementia aetiology are associated with transition times. In addition, we aimed at estimating time from NH admission to death.

## Materials and methods

### Study population

The study population comprised 2,938 home-dwelling persons (57% women) with a clinical diagnosis of dementia during 2008–2017 and registered in the Norwegian Registry of Persons Assessed for Cognitive Symptoms (NorCog) [1,39]. This registry includes persons referred for a dementia workup at outpatient clinics in specialized healthcare across Norway. The persons in the registry have been examined using a standardized examination protocol comprising a large set of cognitive tests (among others the Mini-Mental State Examination (MMSE), the Consortium to Establish a Registry of Alzheimer’s Disease (CERAD), 10-item word list and figure copying, the Clock Drawing Test (CDT), and the Trail Making Tests A and B), an evaluation of activities of daily living (both P-ADL and I-ADL, and IQCODE), a physical examination, CT or MRI of the brain and, in some cases, lumbar puncture for measuring cerebrospinal dementia biomarkers, DaTscan (Ioflupane I 123 injection), 18F-2-fluoro-2-deoxy-D-glucose positron emission tomography (FDG-PET) and 18F-Flutemetamol PET. Including all available information from the workup, the treating physician diagnosed the dementia syndrome and etiological dementia diagnoses based on the ICD-10 criteria for research [40].

### Home address histories, emigration and vital status

Participants were followed for a median of 4.1 years and for a maximum of 10.6 years from time of diagnosis until death, emigration, or until 30 August 2019, whichever occurred first. Information on participants’ address histories and vital status was retrieved from the National Population Registry and linked to the participants by means of the unique personal identification number.

Date of NH admission was identified using three different approaches. First, the study participants’ home addresses were matched with a pre-existing list of NH addresses using the “white pages” (www.1881.no). Second, home addresses were matched with a list of addresses that were likely to be NHs, and the suggested addresses were investigated to determine if they were, in fact, NHs using “white pages” (www.1881.no). Third, we inspected all addresses where the mail was re-addressed to a proxy person. The final address list comprised 325 different NHs covering all Norwegian counties. In Norway, the date for moving to a new address, for instance to a NH, is registered in the National Population Registry within ~20 days after moving, and the final address list was used to assess dates of first NH admission for the study population.

### Covariates

We included the following covariates: sex, marital status (living alone, cohabitating), education (< = 9 years [primary], 10–12 years [secondary], 13+ years [tertiary]), score on the Mini-Mental Status Examination (MMSE) (divided into three severity groups: <19, 19–23, 24–30) [41], and dementia aetiology as follows: Alzheimer’s dementia (AD), vascular dementia (VaD), mixed AD/VaD, dementia with Lewy bodies (DLB), Parkinson’s disease dementia (PDD), other dementias (OD).

### Statistical methods

When studying the amount of time that elapses from diagnosis to NH admission, death is a competing risk that should not be ignored. Persons with dementia dying before NH admission will be censored in standard survival analysis, such as Cox regression. However, an important assumption is that this censoring is independent of health status [42] and, thus, assumes that the censored person is representative of those still at risk. This assumption might hold if respondents are censored at random but is not fulfilled if the person with dementia is censored due to death, such as in the current setting.

**Fig 1:**
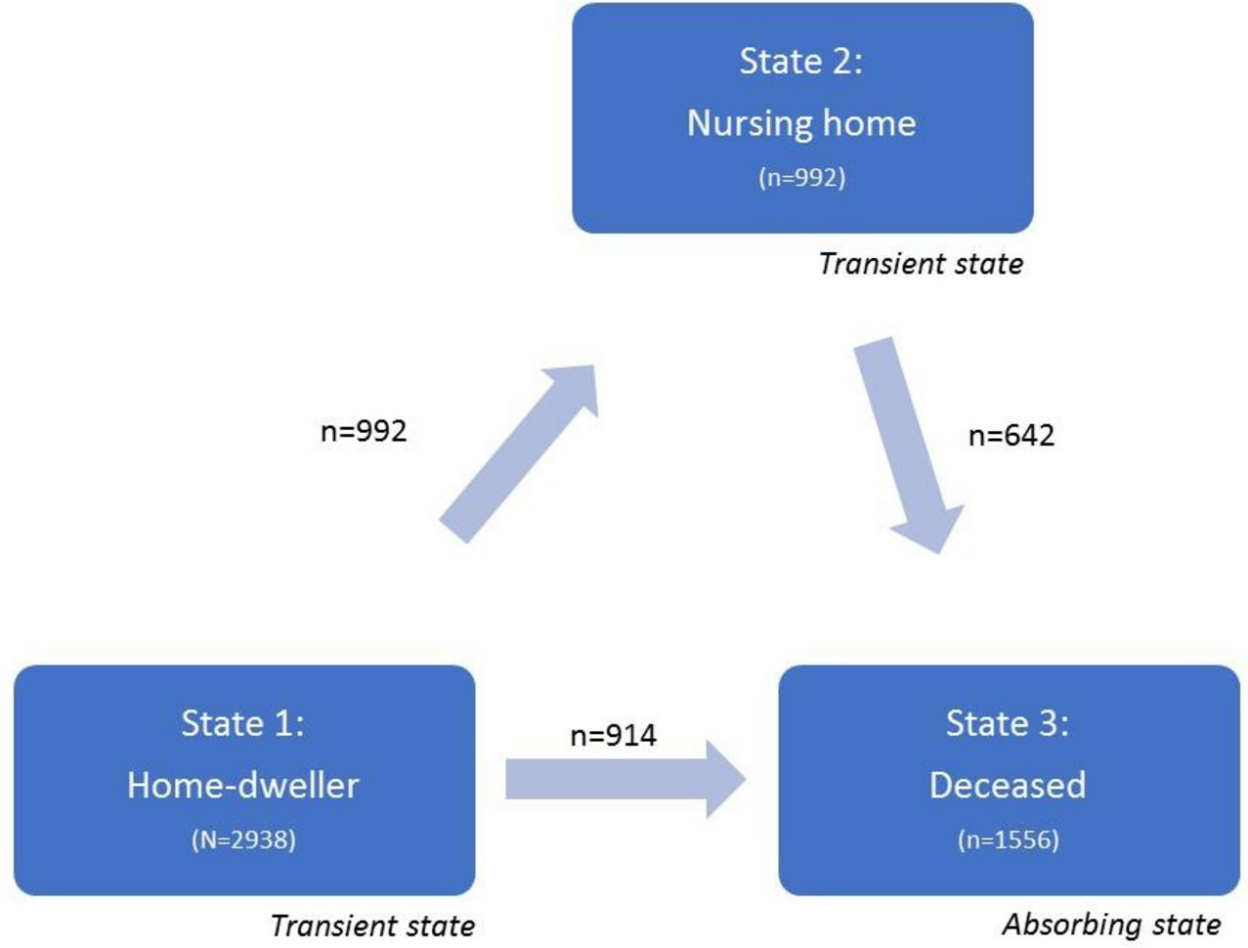
Illness-death model for nursing-home admission. Each transition is a survival model, and the stage the person is in impacts the probability of where to go next.

Therefore, to account for this competing risk, we applied Markov multistate survival analysis (illness-death model) and flexible parametric models. Participants were left-censored on the date of emigration if they emigrated (n = 6) and right-censored on 30 August 2019 if still alive at this date. In survival analysis, we often concentrate on the time to *a single* event, while in practice there might be a variety of intermediate events. In our setting, we followed the person with dementia from diagnosis to NH admission and/or death. Each transition is a survival model, and the stage the person is in impacts the probability of where to go next (Fig 1). As in standard survival analysis, in the multistate survival analysis we can investigate covariate effects for each specific transition between two states. We used the multistate survival package in Stata (*multistate*, *merlin*) [43,44]. Data were reshaped from a wide format (one row of data per person) to a stacked format, where each person had one row of data for each transition for which they were at risk [43]. In the multistate model, we fitted three separate Royston-Parmar models, one for each transition, with three degrees of freedom and time-dependent covariates. Based on these models, we calculated transition probabilities when all participants started out as home-dwellers at the time of diagnosis (time 0). Thus, unlike most survival analyses, which usually report hazard ratios on the relative scale, we present absolute probabilities. In addition to the multistate survival analyses, we applied the t-test and Chi-squared test to assess for differences in mean values and differences in proportions. The significance level was set to 5%.

#### Ethics

This project is approved by the Regional Committees for Medical and Health Research Ethics (2015/1510/REK vest) and by the registry owners (NorCog, Norwegian Institute of Public Health and The Norwegian Tax Administration). NorCog is a consent-based registry, meaning only people who are considered capable of giving an informed consent are included. Persons in early stage of dementia usually can provide informed consent, and as the majority have mild stage dementia at time of diagnosis few people are excluded/not included. All persons included in the registry have given written informed consent for the information from the diagnostic assessment to be stored until 2030 and used for research.

## Results

All persons with dementia were home-dwelling at the time of diagnosis and had the cognitive capacity to give informed consent (N = 2,938). During follow-up, there were four possible pathways for participants; survived as home-dwellers (35%); moved to a NH and survived (12%); moved to a NH and died (22%); or died as home-dwellers (31%) (Fig 1). The median amount of time as a home-dweller before either moving to a NH or dying was 3.29 years (IQR 2.73). For those who moved to a NH, the median amount of time until NH admission was 2.28 years (IQR 2.32), and then the median time from NH admission to death was 2.30 years (IQR 2.32). Overall median survival time from time of diagnosis was 4.06 years (IQR 2.92).

Baseline characteristics differed for men and women. Women were older than men (76.6 years [SD 8.6] versus [vs] 75.2 years (SD 8.4), t-test for difference p<0.01); women more often lived alone than men (58% vs 22%, chi-test for difference p<0.001); women had poorer performance on the MMSE compared with men (<23 on MMSE: 28% in women vs 23% in men, p<0.001); and women had less education compared with men (49% vs 36% with <10 years (primary) education, p<0.001).

Table 1 presents number of participants at baseline admitted to nursing homes and deceased during follow-up by covariates.

**Table 1:** Background table. Number of participants at baseline, and the number of these admitted to nursing homes and/or deceased during follow-up, by covariates.

	Number of participants at baseline (home-dwellers)	Number who entered NHs during follow-up	Number of deceased during follow-up
Total	2938	992	1556
Sex			
Women	1679	656	830
Men	1259	336	726
Age			
<65	297	80	101
65–69	337	98	131
70–74	503	145	241
75–79	683	209	351
80–84	658	264	401
85+	460	196	331
Marital status (#missing values:108)			
Cohabitant	1622	432	847
Living alone	1208	529	652
Education (#missing values:236)			
Low (<10 years)	1177	389	655
Middle (10–12 years)	759	262	377
High (13+ years)	766	250	377
Baseline MMSE (#missing values:47)			
<19	755	267	464
19–23	1235	461	653
24–30	901	249	416
Aetiology			
AD	1549	501	732
VaD	269	75	167
Mixed AD/VaD	433	169	258
DLB/PDD	227	71	146
Other dementias	460	176	253

MMSE: Mini mental state examination, AD: Alzheimer’s dementia, VaD: Vascular dementia, DLB: Dementia Lewy Body, PDD: Parkinson disease dementia.

### Time to nursing-home admission, unadjusted analyses

**Fig 2:**
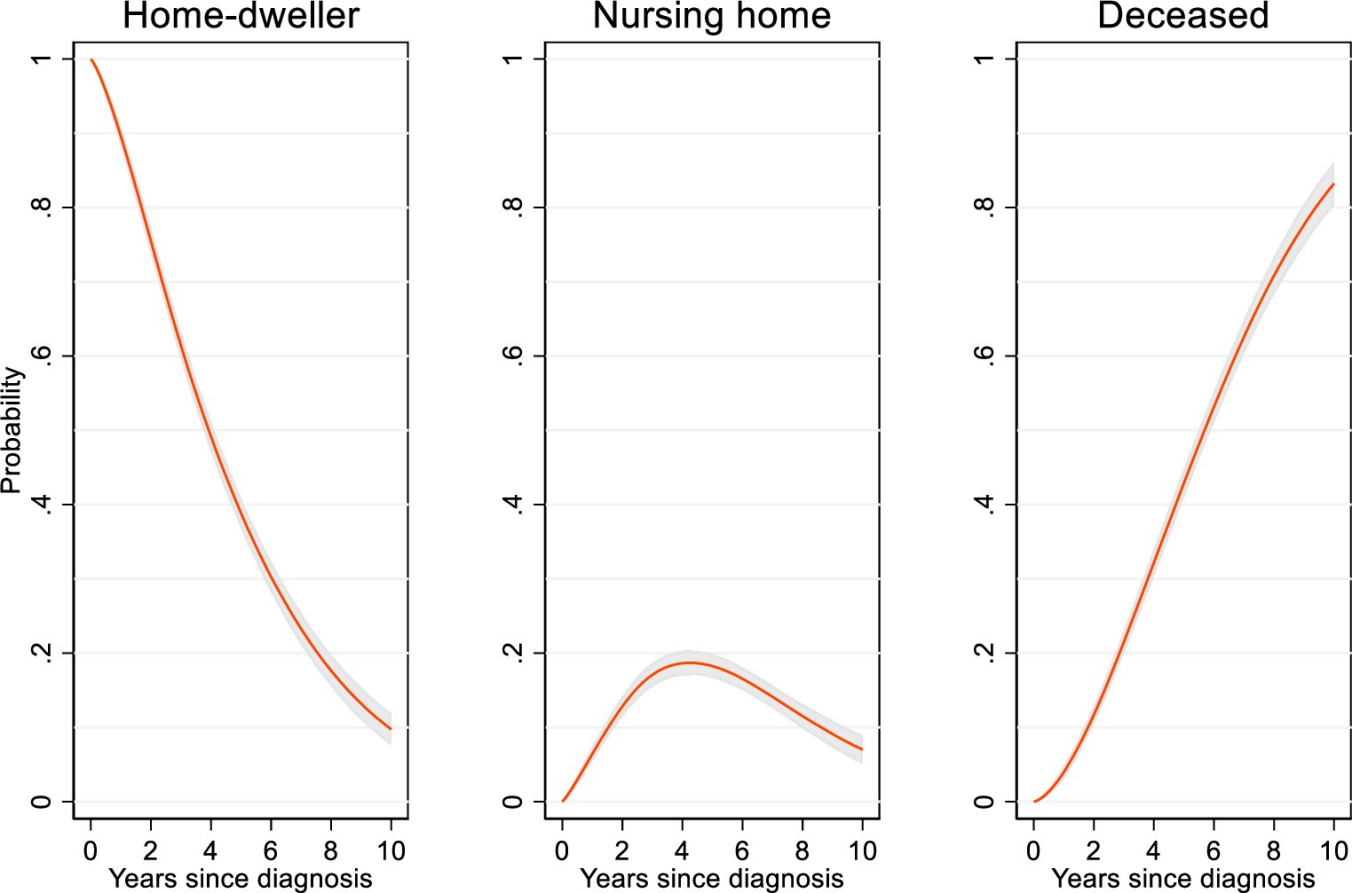
Probability of being either a home-dweller, nursing-home resident or deceased during a 10-year follow-up after dementia diagnosis. Unadjusted analysis, N = 2938. The shaded area is a 95% confidence interval.

In a crude analysis without covariate adjustment, which shows the actual transition times for the persons with dementia in NorCog, the probability of remaining a home-dweller decreased steadily over time, due to both NH admission and death (Fig 2). Simultaneously, the probability of being in a NH increased steadily over time and peaked about four years after diagnosis, after which the probability decreased due to increased mortality (Fig 2). Four years after diagnosis, the probability of still living at home was 49%, while the probability of being in a NH peaked at 19% (95% confidence interval [CI] 17, 20) (Fig 2). Six years after diagnosis, 16% still lived in a NH, while after eight years 11% were still NH residents. After 10 years, 10% lived at home, 7% were NH residents, and 83% had died (Fig 2). There was a slightly higher probability (five percentage points) of NH admission in the oldest age group (75+) compared to the youngest group (<65 years).

### Time to nursing-home admission, adjusted analyses

Focusing on differences across covariate values, analyses were adjusted for sex, age, marital status, education and type and severity of dementia.

### Sex

Men had a lower probability of NH admission than women; four years after the dementia diagnosis, men had a significantly lower probability of being in a NH at seven percentage points compared with women (Table 2). This sex difference was due to the significantly higher male mortality rate.

**Table 2:** Covariate difference in probability percentage points (95% confidence interval, CI) for being at one of the three stages, 4 years after dementia diagnosis.

	Model 1. Unadjusted, N = 2589			Model 2. Fully adjusted for all included covariates in table, N = 2589		
	Home-dweller	Nursing home	Deceased	Home-dweller	Nursing home	Deceased
Constant (Ref)*				40	15	46
sex						
Women	Ref	Ref	Ref	Ref	Ref	Ref
Men	0 (-4, 4)	-9 (-12, -6)	9 (5, 13)	-11 (-15, -7)	-7 (-10, -3)	18 (13, 22)
Age (years)						
<65	30 (25, 36)	-5 (-10, -1)	-25 (-29, -21)	29 (23, 35)	-3 (-6, 1)	-26 (-32, -20)
65–69	24 (19, 29)	-1 (-6, 4)	-23 (-28, -18)	22 (17, 28)	2 (-2, 6)	-25 (-30, -19)
70–74	17 (12, 21)	-5 (-8, -1)	-12 (-16, -8)	14 (9, 19)	-2 (-5, 2)	-12 (-17, -6)
75+	Ref	Ref	Ref	Ref	Ref	Ref
Marital status						
Cohabitant	Ref	Ref	Ref	Ref	Ref	Ref
Living alone	-14 (-18, -10)	11 (8, 14)	3 (0, 6)	-10 (-15, -6)	9 (6, 13)	1 (-3, 6)
Education (years)						
Low (≤9)	Ref	Ref	Ref	Ref	Ref	Ref
Middle (10–12)	5 (0, 9)	0 (-2, 3)	-6 (-9, -3)	-1 (-6, 3)	4 (1, 7)	-2 (-7, 2)
High (13+)	8 (3, 12)	-1 (-3, 2)	-7 (-10, -4)	-2 (-7, 3)	4 (0, 7)	-2 (-6, 3)
MMSE						
<19	Ref	Ref	Ref	Ref	Ref	Ref
19–23	11 (6, 16)	0 (-3, 3)	-11 (-15, -7)	12 (8, 17)	1 (-2, 4)	-13 (-17, -9)
24–30	21 (16, 26)	-6 (-9, -3)	-15 (-19, -12)	20 (15, 25)	-3 (-6, 0)	-17 (-21, -13)

* Probability for the combination of reference values: women, age 75+ years, cohabitant, less education (<10 years), MMSE <19. MMSE: mini mental state examination.

### Age

NH admissions were similar across age at diagnosis.

### Marital status

Persons living alone had a higher probability of NH admission than cohabitants (Table 2). However, the size of this marital-status effect differed between the sexes; there was a significant interaction between sex and marital status (Table 3). Marital status had a significantly larger impact on NH admission among men than among women. Adjusted by age and centred at age 75 years, men living alone had a 24% probability of living in a NH four years after the dementia diagnosis compared to only 11% for cohabitating men, and the difference was significant (p<0.05) (Table 3). This difference was not due to survival because men who lived alone had significantly poorer chances of survival compared with cohabitants. For women, probabilities of NH admission also differed significantly between those who lived alone (26%) and cohabitants (20%), but the difference across marital status was significantly smaller than in men.

**Table 3:** Probability of being either a home-dweller, nursing home resident, or deceased four years after the dementia diagnosis, by sex and marital status, adjusted by age (centred at mean age 75 years), N = 2830.

	Probability, % (95% confidence interval)		
Time elapsed since diagnosis	Home-dweller	Nursing home	Deceased
Men			
Living alone	37 (32, 43)	24 (20, 29)	38 (34, 43)
Cohabitant	54 (51, 57)	11 (10, 13)	35 (32, 38)
*Difference in probability*: P(live alone)-P(cohabitant)*	*-16 (-22*, *-11)*	*13 (9*, *17)*	*3 (-2*, *9)*
Women			
Living alone	50 (47, 53)	26 (23, 29)	24 (21, 27)
Cohabitant	55 (52, 59)	20 (17, 23)	25 (22, 27)
*Difference in probability*: P(live alone)-P(cohabitant)*	*-6 (-11*, *-1)*	*6 (3*, *10)*	*0 (-5*, *3)*

*In some cases, numbers do not add up due to rounding.

### Education

Persons with 10 or more years of education were significantly more likely to be admitted to a NH compared with those with nine or fewer years of education. The difference in probability of being in a NH after four years was four percentage points. The higher likelihood of NH admission in the more-highly educated groups was not entirely explained by lower mortality.

### Severity of cognitive impairment and type of dementia

Severity of cognitive impairment, measured at baseline with the MMSE, did not impact NH admission significantly after adjusting for age, sex, marital status and education. The probability of being a home-dweller, however, was positively associated with MMSE score, due to the lower mortality rate for those with higher MMSE scores.

Type of dementia did not impact the probability of living in a NH after four years, except for the group “other dementia”, which had a slightly higher probability of five percentage points compared to AD (Table 4). Yet, type of dementia did have an impact on mortality; significantly more persons with AD still lived at home and fewer were dead after four years than persons with any other types of dementia.

**Table 4:** Diagnosis difference in probability (95% confidence interval, CI) for being at the three stages 4.0 years after dementia diagnosis, adjusted by age, sex, MMSE, education.

	Probability, % (95% CI)		
	Home-dweller	Nursing home	Deceased
Constant (Ref)*	48	22	30
Diagnosis			
AD	Ref	Ref	Ref
VaD	-13 (-19, -6)	-2 (-7, 3)	15 (9, 21)
Mixed	-6 (-11, -1)	2 (-2, 6)	4 (-0, 9)
DLB/PDD	-17 (-23, -10)	-2 (-7, 4)	18 (11, 25)
Other	-10 (-15, -5)	5 (0, 9)	5 (1, 10)

*Predicted at age 75 years, for women, MMSE<19, less education (<10 years). MMSE: mini mental state examination, AD: Alzheimer’s dementia, VaD: vascular dementia, DLB: Dementia Lewy Body, PDD: Parkinson disease dementia.

### Survival in a nursing home

**Fig 3:**
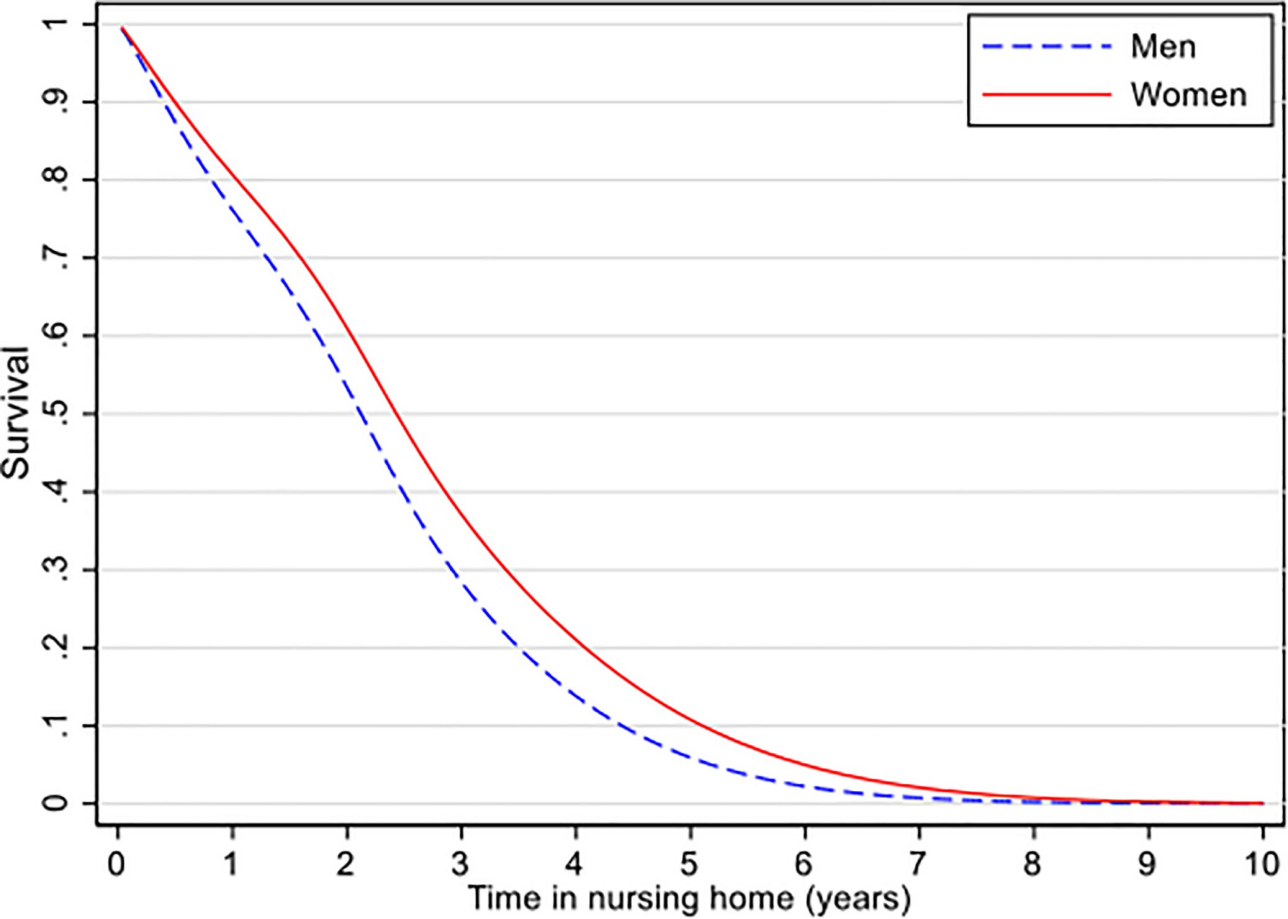
Survival in nursing home by sex. N = 992, estimated in flexible parametric models.

Among the 993 patients admitted to NHs, the median survival time was 2.1 years (inter quartile range (IQR) 2.1) for men and 2.4 (IQR 2.4) years for women. Of the persons admitted to NHs, 80 percent had died after 3.5 years among men and after 4.0 years in women (Fig 3).

## Discussion

In this large specialist healthcare population of persons with dementia, the probability of being a NH resident peaked approximately four years after receiving the dementia diagnosis for all age groups; thereafter, the probability decreased due to mortality. Women had a higher probability of NH admission than men, due entirely to their lower mortality rate. Furthermore, persons living alone, especially men, had a higher probability of NH admission than cohabitants. Severity of cognitive impairment and type of dementia did not influence the probability of NH admission but did influence probability of mortality. Having less education was associated with lower NH admission independent of mortality.

In this study, where the participants had been diagnosed in specialist healthcare, no difference was found in the time to NH admission between age groups. A Dutch study found that older age was a strong predictor for shorter time to institutionalization [14]. In Norway, assessing and diagnosing persons older than 65 years with symptoms of cognitive decline is mainly a primary healthcare responsibility [8]. Persons diagnosed in primary healthcare are typically older, less educated, and have poorer cognition and more limitations in ADL than persons diagnosed in specialist healthcare [7,9]. Unfortunately, we do not know if all the persons included in other studies were diagnosed in specialist healthcare or also in primary healthcare [14,17,18].

After four years, fewer men than women still lived at home. This was due entirely to the higher male mortality rate because the NH admission rate was lower in men than in women.

Studies have shown no consistent effects of marital status or living arrangements on NH admission [6,26,31], even though there are studies providing support to the hypotheses that living alone and being a widow(er) or having a non-spousal informal caregiver increases the risk of NH admission [18,27,28]. In our study, living alone significantly increased the probability of being admitted to a NH for both men and women, but the association was especially strong for men. This excess probability among persons living alone was seen even though mortality rates did not differ significantly from those of cohabitants. Men with a spouse seldom moved to a NH, and therefore, the reasons for cohabitants not being home-dwellers were largely ascribed to mortality. In women, however, the reasons for cohabitants not being home-dwellers were ascribed equally to NH admission and mortality. Unfortunately, the reason for this sex by marital status interaction cannot be investigated further in our data, but we may speculate that women provide care at home for the male spouse to a larger extent than men do for their spouses. There may be a number of reasons for this, with age being one. Women often marry older men, who, being older, have more frailty and impairment; hence, they are less able to provide care and support at home for their wives [15,29,30].

In line with the review by Cepoiu-Martin and colleagues [27], we found that type of dementia disorder did not predict NH admission. We did find, however, that persons with AD had a higher probability of continuing to live at home four years after the diagnosis than persons with other types of dementia; however, this was due entirely to higher survival rates in AD, as also reported earlier [1]. In contrast to Belger et al [18] who found that MMSE baseline severity was associated with institutionalisation, we found that worse performance on the MMSE, which is indicative of greater cognitive impairment, did not predict NH admission but only death. This may indicate that the baseline MMSE score does not necessarily yield information about the degree of impairment in ADL or changes in behaviour. Whether or not a person is able to remain at home is probably associated with available formal and informal support, ADL function, behaviour, physical function and his or her number of comorbidities [6,30].

Surprisingly, those with 10 or more years of education had an increased probability of being admitted to a NH compared to those with nine years or less. This excess probability was not due to confounding by age or gender or due to mortality differences between the educational groups. As healthcare is public, and there is no difference in admission to public vs private nursing homes in Norway, why there is a difference in probability of NH admission between the education-attainment groups is puzzling, although persons with more education have been found to be prone to utilize more healthcare [37]. A German study showed an increased use of NHs when the relatives of the NH resident had higher education. This was associated with economic resources like out-of-pocket costs and having nursing care insurance [30]. It may be that persons with higher education have more knowledge about their rights to public healthcare service as well as the resources to acquire these services, in addition to having relatives to help them secure public services provided by law.

### Survival in NHs

The Danish study by Reilev and colleagues found that men in NHs have a higher prevalence of comorbidities [15] and shorter survival compared to women, a finding that aligns with our results. As both sexes receive the same type and amount of care in the NH, it is difficult to understand why men have shorter survival in a NH. One explanation could be that men experience a more rapid ageing process compared to women. Previous research has demonstrated that men with dementia have a shorter survival rate compared to women [1,13,14].

#### Strengths and limitations

In Norway, no registry of NHs exists. Thus, some NH admissions may have been wrongly included or excluded due to difficulties finding the addresses or exact dates of admission. Nevertheless, we believe this to occur at a similar rate across covariate groups, and therefore, we trust the observed differences in NH admission to be accurate. As persons diagnosed in the specialist healthcare differ from those diagnosed in primary healthcare, the results might not be representative for all persons with dementia.

A key strength of the study is the large number of participants included from hospitals across the country. In addition, they were diagnosed using the standardized diagnostic assessments performed in the hospitals, thereby ensuring a valid dementia diagnosis assessment. Moreover, access to the NorCog data allowed us to combine key national registries and was of great value for the study.

## Conclusion

The new knowledge on time from diagnosis of dementia to NH admission and key variables associated with this transition time can be used to inform individuals, their families and policy makers about transition times and progression of the different dementia disorders. Knowledge about transition times allows the healthcare and social services to plan for provision of the appropriate services at the right time to the person with dementia so that he or she can live at home for as long as possible. Half of the persons with dementia still lived at home four years after diagnosis, and NH admission peaked at 19%. Men had a lower probability of being admitted to NHs than women due to higher mortality, and having a partner was associated with a lower probability of NH admission, especially in men. As healthcare is public and accessible to all persons in Norway, a lower probability of NH admission for persons with less education is alarming, and the difference in the probability of NH admission between the groups in relation to educational attainment should be further studied.

*Transparency declaration*: The lead author* affirms that this manuscript is an honest, accurate, and transparent account of the study being reported; that no important aspects of the study have been omitted; and that any discrepancies of the study as planned have been explained.

*Public and patient involvement* was provided prior to this investigation by the user council at the Norwegian National Advisory Unit on Ageing and Health, and with the cooperation of the Norwegian Health Association, the user organization for persons with dementia in Norway.

**Summary box**

**Section 1: What is already known on this topic**

- Few studies have examined the exact amount of time from the dementia diagnosis to nursing-home admission or death.

**Section 2: What this study adds**

- Four years after diagnosis, nursing-home admission peaked at 19%.
- No differences were found in the probability of nursing-home admission related to different aetiological dementia diagnoses.
- Less education was associated with a lower probability of NH admission, independent of mortality.
